# Effects of Vitamin D on Fertility, Pregnancy and Polycystic Ovary Syndrome—A Review

**DOI:** 10.3390/nu14081649

**Published:** 2022-04-15

**Authors:** Szabolcs Várbíró, István Takács, László Tűű, Katalin Nas, Réka Eszter Sziva, Judit Réka Hetthéssy, Marianna Török

**Affiliations:** 1 Department of Obstetrics and Gynecology, Semmelweis University, Üllői Street 78/a, 1082 Budapest, Hungary; varbiro.szabolcs@med.semmelweis-univ.hu (S.V.); torok.marianna@med.semmelweis-univ.hu (M.T.); 2 Workgroup of Research Management, Doctoral School, Semmelweis University, Üllői Street 22, 1085 Budapest, Hungary; hetthessy.judit_reka@med.semmelweis-univ.hu; 3 Department of Internal Medicine and Oncology, Semmelweis University, Sándor Korányi Street 2/a, 1098 Budapest, Hungary; takacs.istvan@med.semmelweis-univ.hu; 4 EndoCare Institute, Endocrinology Center, Bokor Street 17-21, 1037 Budapest, Hungary; tuul68@gmail.com (L.T.); drnaskatalin@yahoo.com (K.N.); 5 Department of Physiology, Semmelweis University, Tűzoltó Street 37-47, 1094 Budapest, Hungary

**Keywords:** polycystic ovary syndrome, PCOS, vitamin D, vitamin D deficiency, vitamin D supplementation, female fertility, male fertility, in vitro fertilization, pregnancy, reproductive health

## Abstract

Polycystic ovary syndrome (PCOS) is one of the most common endocrine reproductive disorders in women. Vitamin D deficiency is also quite common in this condition. The degree of vitamin D deficiency correlates with the severity of PCOS. Both male and female vitamin D levels play a role in fertility and affect the outcomes of in vitro fertilization (IVF). Moreover, fertility and IVF indicators are improved by vitamin D not only in healthy women but in those diagnosed with PCOS. Both vitamin D deficiency and PCOS increase pregnancy-related complications. Vitamin D supplementation and optimal vitamin D levels decrease both maternal and fetal risk for complications and adverse events. Furthermore, vitamin D supplementation may ameliorate or even prevent pregnancy-related reversible bone loss in mothers. This review emphasizes the roles of vitamin D deficiency and vitamin D supplementation and their correlation with PCOS regarding reproductive health.

## 1. Introduction

Polycystic ovary syndrome (PCOS) is one of the most common endocrinological conditions in reproductive-age women. PCOS decreases the chances of conception and increases the risk of complications during pregnancy. This syndrome in often accompanied by vitamin D deficiency, which occurs in 67–85% of cases [1]. Several studies have shown that vitamin D deficiency is linked to chronic diseases, such as endocrinological or tumorous conditions and autoimmune diseases among others [2,3]. It has also been described that adequate vitamin D supplementation may improve the condition of these patients [4]. Vitamin D interacts with the epigenome on multiple levels—this regulates approximately 3% of the human genome [5]. Critical genes in the vitamin signaling system have large cytosines followed by guanine (CpG) islands—these influence DNA methylation [6,7,8]. The vitamin D receptor (VDR) interacts with numerous co-activator and co-repressor proteins, while chromatin modifiers and remodelers are VDR and related ligand targets [9]. Alterations in DNA methylation lead to both aberrant gene expression genomic integrity disruptions, resulting in the development and progression of several diseases. Vitamin D is involved in regulating epigenetic processes and is in turn regulated by epigenetic mechanisms [6]. The epigenetic modifications themselves are reversible via the CYP2R1 microsomal 450 enzyme, which is hydroxylated at position C-25 in D2 and D3. When vitamin D levels are low, the 25-hydroxylase CYP2R1 promoter may be methylated. This process may be reversed by vitamin D supplementation [6,10]. One of the numerous biomarkers for determining biological age is DNA methylation age—marking deviation from chronological age with DNAm (age acceleration—DNAmAA) [11,12]. DNAmAA was found to be 2.6 years lower in those who were vitamin D deficient before but received vitamin D supplementation compared to those without supplementation [13]. Obesity, PCOS and pregnancy increase vitamin D demand; thus, vitamin D deficiency is more common and more severe in these conditions compared to healthy individuals [14,15]. The results of the current research have shown that vitamin D plays a role in both male and female fertility; therefore, it is of paramount importance to keep vitamin D levels in the optimum range in case of fertility-related conditions, i.e., PCOS. In our current study, we summarize the current knowledge of the effects of vitamin D on fertility, pregnancy and pregnancy-related complications in PCOS and other gynecological conditions.

## 2. Materials and Methods

In this review, we provide an overview of the potential roles of vitamin D in fertility, in in vitro fertilization (IVF), and in pregnancy in healthy and PCOS women. We also analyze the possible influence on certain bone-related obstetric–gynecological conditions. This work is based on analysis of the literature—a PubMed search up to September 2021 using at least one of the following search terms: vitamin D, vitamin D deficiency, polycystic ovarian syndrome, PCOS, fertility, in vitro fertilization, pregnancy, pregnancy complications. Systematic reviews, meta-analyses and review articles were included in our research. To complement our article base, we also added select relevant articles from the references of these articles and publications used in our previous work. Animal studies were also discussed if they were deemed relevant. We did not limit our research to specific results but aimed to provide an up-to-date overview of the effects of vitamin D deficiency and supplementation on fertility, IVF, and pregnancy in healthy and PCOS subjects. We also summarize recommendations for the clinical management of vitamin D treatment.

## 3. Influence of Vitamin D on Fertility and In Vitro Fertilization

The key role of vitamin D regarding fertility is emphasized by the fact that vitamin D receptors (VDRs) present in both male and female central and peripheral reproductive organs, tissues and cells. VDRs are also found in the hypothalamus, the hypophysis, in the ovaries, granulosa cells, the endometrium, the placenta, the decidua and in the testes and the cells of spermatogenesis in men [3,16]. The Pit-1 gene in the hypothalamus carries a VDR promoter, which plays a significant role in the development of the anterior hypophysis and the activation of both growth hormone (GH) and the prolactin gene [17]. Increasing the production of ovarian steroid hormones—progesterone, estradiol and estrone—and follicular stimulation hormone (FSH) receptor genes, as well as follicular maturation and selection are some of the physiological effects of vitamin D [18]. Anti-Müllerian hormone (AMH) levels—which are good indicators of ovarian function—also correlate with vitamin D levels and accordingly show seasonal changes: AMH level decreases by 18% in the winter compared to summer values; the change in AMH level correlates with the magnitude of change in initial AMH and vitamin D levels [19]. These changes are similar to those seen regarding pregnancy rates [20].

Animal experiments demonstrate that vitamin D plays an important role in both male and female reproductive functions. Reproductive function was damaged in various ways in VDR-knockout females: uterine hypoplasia, decreased aromatase activity and aromatase gene expression occurred. Serum luteinizing hormone (LH) and follicular stimulation hormone (FSH) levels were increased, meaning that the primary damage appeared in peripheral tissues (i.e., hypergonadotropic hypogonadism) [17,21]. Similar to female VDR-knockout mice, reproductive functions were also impaired in males: lower sperm count, motility and histological abnormalities in the testes were observed, while testosterone levels remained physiological [17,21]. The results of human studies showed similarities to animal experiments, as males with sufficient vitamin D levels have better spermiogram results than men with vitamin D deficiency (levels below 20 ng/mL). More fertile men demonstrated higher vitamin D levels (21 ± 10 vs. 16 ± 9 ng/mL) [22]. In the case of males, vitamin D may have an age-dependent effect on testosterone. There is no correlation between testosterone, LH and vitamin D levels in young adolescents, while in older men, a positive association was found between vitamin D and testosterone levels [17]. Maternal vitamin D level has an effect on the selection of the ‘best sperm’ during the fertilization process, facilitating appropriate capacitation, hyperactivation and acrosomal reaction. However, paternal vitamin D levels do not have an effect on these processes, as there is no vitamin D present in the semen [23].

Vitamin D level influences the outcomes of in vitro fertilization (IVF) independently from age, body mass index (BMI), ethnicity and the number of embryo transfers [17,24]. Vitamin D levels correlate strongly with success rates (r = 0.94); every nmol/L increase in the vitamin D content of the follicular fluid increases the probability of clinical pregnancy by 2.4% [24]. Vitamin D increases the survival of the preantral follicle, sustains AMH production and enhances the growth of the antral follicle [25]. It also affects the length of telomeres and telomerase enzyme activity, thereby decreasing the aneuploid ratio and improving the efficacy of IVF treatment [16].

In summary (Table 1), we may state that optimal vitamin D levels in both males and females play an important role in fertility and the outcomes of IVF.

## 4. Polycystic Ovary Syndrome and Vitamin D Deficiency

Prevalence of PCOS is 5–20% in reproductive-aged women [26]. Differences in diagnostic criteria contribute to a wide range of prevalence values: prevalence based on the National Institute of Health (NIH) criteria, 8.7 ± 2.0%; according to Rotterdam criteria, 17.8 ± 2.8%; while prevalence is 12.0 ± 2.4% based on the criteria of the Androgen Excess Society (AES) [27]. PCOS—characterized by clinical and/or biochemical hyperandrogenism, menstrual disorders such as oligomenorrhea/amenorrhea and ovarian dysfunction, polycystic ovary morphology—is one of the most frequent reasons of infertility among reproductive-aged women [28]. The root cause of PCOS has not yet been established; it is now considered to be multifactorial where both genetic and environmental factors play a role. There is cumulative evidence suggesting that insulin resistance also plays a key role [29,30]. As we mentioned above, vitamin D deficiency is quite common in this patient group (67–85%) and may appear in a more severe form although the single-nucleotide polymorphism (Apa I, VDR Fok I, Taq I) of the vitamin D receptor (VDR) does not correlate with the risk of developing PCOS or with the severity of the disease [31,32]. Vitamin D status in PCOS is linked to reproductive function, metabolic alterations and mental health [33].

Vitamin D therapy decreases serum androgen and AMH levels in PCOS patients, and it decreases endometrial thickness [34]. In combination, they have positive effects on menstrual cycle and on folliculogenesis [33]. Menstrual irregularities such as oligomenorrhea and amenorrhea are quite common in PCOS (found in 38% of the cases) [27]. Ninety percent of patients suffering from amenorrhea are diagnosed with PCOS [35]. The probability of ovulation correlates with vitamin D levels in PCOS, showing 68% probability below levels of 20 ng/mL, 77% between 20–30 ng/mL, and 78% above 30 ng/mL [28]. Pathologies appear not only in the ovaries of PCOS patients, but in the uterus as well. In animal models, the endometrium, epithelium and stroma were all pathologically thickened, and the thickening was accompanied by increased proliferation and apoptosis. As a result of vitamin D supplementation (weekly doses of 120 ng 1,25(OH)_2_-vitamin D/100 g in a rat model), the thickness of the endometrium and the stroma decreased significantly as did the degree of pathological proliferation and apoptosis [34]. Pathological endometrial structure hinders implantation, while the improved structures induced by vitamin D supplementation facilitate implantation, thereby enhancing fertility indicators. Vitamin D effects endometrial receptivity: women demonstrating increased VDR expression in the endometrium, especially during the implantation of the embryo, were more likely to become pregnant than their counterparts with decreased VDR expression [36]. Endometrial receptivity improved as a consequence of vitamin D supplementation in PCOS patients as well [37].

VDR expression of the granulosa cells and vitamin D content of the follicular fluid are decreased in PCOS, which also suggests that vitamin D supplementation may aid the treatment of infertility in PCOS [38]. In a recent publication, IVF treatment of women suffering from PCOS was complimented with vitamin D supplementation. It was found that both implantation and occurrence of clinical pregnancy were significantly higher in patients with normal vitamin D levels compared to those with decreased levels of vitamin D (<20 ng/mL 25(OH)-vitamin D); vitamin D levels correlate strongly with the likelihood of implantation and clinical pregnancy (*p* < 0.01); they improve embryo quality—the number of high-quality embryos following vitamin D treatment equals that occurring in women with normal vitamin D levels [39].

Insulin resistance may be responsible for the higher rate of miscarriages in PCOS [38,40,41,42]. Vitamin D supplementation improves insulin resistance [3,43,44,45,46] and the quality of embryos, therefore leading to a higher ratio of clinical pregnancies in PCOS [39].

Following ovulation–induction treatment, a lower ratio of live births was found in PCOS patients with low vitamin D levels (25(OH)-vitamin D < 75 nmol/L); live births were lower by 44% (OR: 0.58). This ratio improved markedly following adequate vitamin D supplementation (in case of levels > 95 nmol/L OR: 1.42; in case of levels > 100 nmol/L OR: 1.51; in case of levels > 112.5 nmol/L OR: 4.46). For every 2.5 nmol/L increase in vitamin D levels, the ratio of live births increased by 2% [28].

A possible underlying cause for the varied detrimental effects of vitamin D deficiency may be the link between vitamin D and the glucocorticoid system. During pregnancy, glucocorticoid levels rise physiologically as they help adapt maternal metabolism. However, overexaggerated glucocorticoid exposition has severe disadvantageous effects on the embryo, leading to placental dysfunction and impaired embryonic growth. It may also lead to an increased risk of cardiovascular and neuropsychiatric diseases in adulthood. Both the placenta and embryonic tissues benefit from glucocorticoid exposition to a certain extent. The enzyme responsible for glucocorticoid degradation decreases in vitamin D deficiency. In a mouse experimental model, vitamin D affected maternal glucocorticoid levels, and deficiency led to an increase in circulating cortisol levels: an increase in the size of adrenal glands—marking the chronic activation of the adrenal gland–hypophysis–hypothalamus axis—may lead to the elevation of embryonic glucocorticoids [47].

The effects of vitamin D may also be explained by immunomodulatory effects, and this may be key. The following alterations may be observed in vitamin D deficiency: Th2 cytokine dominance instead of Th1 and decreased proliferation of inflammatory cytokines [48]. Vitamin D inhibits the production of tumor necrosis factor-alpha (TNF-α), interleukin-6 (IL-6) and interferon-gamma (IFN-γ), and increases the production of human chorionic gonadotropin (hCG) in in vitro trophoblast experiments [49]. Its anti-inflammatory activity was demonstrated in trophoblast cultures contaminated by Escherichia coli bacteria—it showed antibacterial effects [47,50].

In summary (Table 2), we may state that Vitamin D deficiency correlates with the severity of PCOS. Fertility and IVF indicators are improved by vitamin D not only in healthy women but in those diagnosed with PCOS, which is important because vitamin D deficiency is both frequent and significant in this patient group.

## 5. Vitamin D Deficiency and Pregnancy

Vitamin D demand increases in pregnancy, 70% of pregnant women suffer from vitamin D deficiency, 21% from vitamin D deficit and only 7.3% show sufficient levels of vitamin D [51]. Vitamin D has effects on decidualization, implantation, expression of human placental lactogen (hPL), secretion of human chorionic gonadotropin (hCG), progesterone and estrogen levels, calcium-uptake in the placenta and the immune responses of the placenta [52]. The risk of individual complications may be increased by PCOS and vitamin D deficiency both independently and linked, while this risk may be decreased by optimal vitamin D levels (>75 nmol/L).

Vitamin D levels have been implemented in most pregnancy-related complications. Vitamin D levels measured in the first trimester (10.1 ng/mL vs. 15.7 ng/mL) correlate with gestational diabetes (GDM) diagnosed later-on during pregnancy in the 24th–26th gestational weeks, independently from conventional risk factors (history of GDM, obesity). A significantly higher percentage of patients with established GDM came from the vitamin D deficiency group, versus the group where vitamin D levels were above 75 nmol/L (87.1% vs. 68.7%) [53,54]. Preeclampsia shows seasonal fluctuation with higher incidences in the winter versus the summer [55], which correlates with the seasonal variability of vitamin D levels. A recent review and meta-analysis confirmed that higher levels of vitamin D during pregnancy were associated with decreased risk of preeclampsia, but further well-designed clinical studies are needed to better explore and define the associations [56,57]. However, a number of large-scale observational studies did not confirm the effects of vitamin D on preeclampsia, and previous studies showed that the risk of preeclampsia does not increase in vitamin D deficiency in healthy pregnant women [58]. Vitamin D enhances Th2-dominance and decreases the expression of certain placental vascular genes (soluble Fms-Like Tyrosine Kinase-1 (sFLT-1 vascular endothelial growth factor (VEGF)), the elevated levels of which have an association with preeclampsia. These factors may play a role in the presumed protective effect of vitamin D [55,59]. Furthermore, vitamin D deficiency is associated with habitual abortion [60,61], and it is more common in women suffering from infertility compared to the normal population [62]. Vitamin D inhibits inflammatory cytokine function (interleukin-2 (IL-2), interferon gamma (IFN-γ) and tumor necrosis factor alpha (TNF-α) may also be key points [63]. Vitamin D levels below 50 nmol/L are associated with an increased risk of miscarriages; however, this correlation was not found in the second trimester [64].

Bacterial vaginosis is often symptom-free; however, it may increase the risk of a variety of complications such as pelvic inflammation, infertility, spontaneous miscarriages and premature births in pregnancy. An inverse association was found between bacterial vaginosis and 25(OH)-vitamin D levels in pregnancy, the risk of bacterial vaginosis increasing in case of vitamin D deficiency [65].

Adequate maternal vitamin D supply has effects not only on fertility and pregnancy complications, but on embryonic complications and the life-long health of the child. Embryonic vitamin D deficiency is partially counterbalanced by the alteration of vitamin D levels during pregnancy as the amount of active vitamin D multiplies independent of calcium, phosphate or parathormone levels [66]. Despite this, a more significant decrease in maternal vitamin D is associated with embryonic complications. The risk of spontaneous premature births increases in maternal vitamin D deficiency. Underlying reasons may include bacterial vaginosis and the fact that vitamin D decreases the production of inflammatory cytokines—as inhibition is blocked and the risk of chorioamnionitis increases as well [55]. The risk of premature birth decreases by 60% when vitamin D levels are above 40 ng/mL [67]. In-utero-experienced vitamin D deficiency increases the risk of juvenile asthma, schizophrenia, sclerosis multiplex, Type 1 diabetes and insulin resistance [52,55]. The risk of Type 2 diabetes increases 1,5-2-fold in children born under vitamin D-deficient conditions, while maternal risk is far greater (especially following GDM) [55]. A recent systematic review confirmed the following hypothesis: during pregnancy, maternal vitamin D deficiency may contribute to the development of autism spectrum disorder (ASD) [68].

The placenta plays a cardinal role in the development of most pregnancy complications such as preeclampsia, intrauterine growth restriction (IUGR), preterm birth, or gestational diabetes mellitus (GDM). The pathomechanisms in the placenta are the result of a wide variety of processes such as oxidative–nitrative stress, vascular malformation, impaired trophoblast invasion, proinflammatory cytokines and the effects of angiogenic growth factors (specifically Placental growth factor (PlGF) and sFlt-1) [69,70,71]. Vitamin D also exerts its protective role regarding pregnancy complications via the placenta. VDR expression of the placenta was decreased in intrauterine retardation [72]. Expression of the mammalian target of rapamycin (mTOR)—serving as a nutritional sensor of the placenta—decreased in VDR knockout mice as well, which had a key effect on embryonic development [73,74]. Furthermore, the expression of amino-acid transporter genes connected to embryonic development also correlates with maternal vitamin D and vitamin-D-binding protein levels. It seems that vitamin D plays a relevant role in the placental transportation of amino acids to the embryo [75]. In case of preeclampsia, the protective effects of vitamin D are multifold. Vitamin D maintains endothelial function by improving proliferation, migration and tubular formation. Vitamin D affects the placenta, placental invasion, implantation and angiogenesis. It also reduces the rate of oxidative stress in the placenta [76,77]. Vitamin D treatment on human fetal endothelial colony-forming cells restored preeclampsia or hypoxia damage via capillary-tube formation and the enhancement of the migration of fetal endothelial colony-forming cells [77]. However, the protective effects of vitamin D regarding preeclampsia are realized not only through the placenta but also through systemic effects. This is especially true in cases of late-onset placental-mediated complications [77]. Regarding preterm birth, the protective effects of vitamin D via the placenta have also been observed. There is an association between the Fok-l and Cdx-2 polymorphism of the VDR gene and spontaneous preterm birth, suggesting that they are involved in the induction of these syndromes [78]. Furthermore, vitamin D may reduce the risk of preterm birth by regulating corticotropin-releasing hormone and other mediators of labor in human syncytiotrophoblasts [79]. Concomitant GDM and vitamin D deficiency increase placental VDR protein and mRNA levels in placental and umbilical cord blood samples [80,81]. Increased expression of the CYP24A1 protein and mRNA—responsible for vitamin D catabolism—is also observed in GDM, which may contribute to the commonality of vitamin D deficiency in GDM [80]. VDR expression is regulated in a bimodal fashion by calcitriol: high doses (0.1 and 1 nmol/mL) lead to downregulation, while low doses (0.01 nmol/mL) lead to VDR upregulation [81]. Vitamin D has a reducing effect on inflammation in the placenta in GDM. Furthermore, in GDM placentas, the bioavailability of VDR-D3 complex increases as a compensatory mechanism [82].

Vitamin D supply of newborns is strongly dependent on maternal vitamin D supplies, and without external supplementation, breast milk is the only source of vitamin D during this period. During breastfeeding, amounts over 4000 IU of maternal vitamin D supplementation lead to a similar increase in 25(OH)-vitamin D levels of the feeding infant as external supplementation [83,84]. 

There is an inverse association between the need for a primary cesarean section and vitamin D status. Women with severe vitamin D deficiency (25(OH)-vitamin D levels <37.5 nmol/L) delivered almost four times as often by cesarean section as those with 37.5 nmol/L or greater levels (OR 3.48) [55,85].

Maternal vitamin D supplementation has a positive effect on birth weight, and it reduced the risk of low birth weight and small-for-gestational-age (SGA) births [86,87]. Furthermore, maternal vitamin D status has an early prenatal effect on fetal skeletal development with a lasting postnatal effect [88,89].

In summary (Table 3), vitamin D deficiency may have the following effects on pregnancy: increased risk for preeclampsia, GDM, spontaneous premature birth and bacterial vaginosis. We may also conclude that vitamin D demand increases in pregnancy: optimal vitamin D levels decrease both maternal and fetal risks.

## 6. Vitamin D Deficiency and Female Bone Mineral Density (BMD) in Childbearing Period and Pregnancy-Related Transient Osteoporosis of the Hip

Another interesting question: do low vitamin D levels influence bone mineral density (BMD) in women at different life periods (e.g., childhood, adolescence, reproductive age, pregnancy, postpartum, menopause), especially during the childbearing years: reproductive age, pregnancy and postpartum puerperium (Table 4)? Unfortunately, there are few studies that investigated the BMD of pregnant and/or postpartum lactating women [90]. Møller UK et al. investigated women’s BMD during pre-pregnancy and pregnancy (in the first, second and third trimesters) and in postpartum. They found that the lumbar spine, total hip, whole body and ultra-distal forearm BMDs decreased significantly during pregnancy; additionally, postpartum BMD decreased further (this may be due to breastfeeding), despite the fact that during pregnancy and postpartum, women had higher daily total intakes of vitamin D compared to controls, although adjustment to vitamin D intake did not change the BMD results. Nineteen months after delivery, BMD did not differ from pre-pregnancy levels, which indicates that pregnancy and breastfeeding may lead to reversible bone loss [91]. Ó Breasail M et al. studied 30–45-year-old healthy pregnant and non-pregnant non-lactating women; their volumetric BMDs (vBMD) in the tibia and the radius were measured with peripheral and high-resolution quantitative CT (pQCT and HR-pQCT) during the 14th–16th and 34th–36th gestational weeks. A significant decrease in vBMD and a different microarchitectural pattern were mainly found in the tibia, but not in the radius in the pregnant group. These results point to a compartment-specific maternal BMD and microarchitectural changes during pregnancy [92]. However, in these studies, 25(OH)-vitamin D levels were not measured; thus, the exact vitamin D supply categories (optimal/suboptimal/deficient) during the investigated period (pre-pregnancy, pregnancy and postpartum) are unknown. MAVIDOS, the MAternal VItamin D Osteoporosis Study—among other things—investigated a maternal bone resorption marker (urinary C-terminal telopeptide of type I collagen/CTX), 25(OH)-vitamin D levels and the effects of gestational vitamin D supplementation (1000 IU/day) on resorption markers and BMD during pregnancy [93]. During the initial 14 weeks of gestation, there was no difference between the 25(OH)-vitamin D levels in the two groups (in nmol/L: placebo group, 44.1 ± 16.1; vitamin-D-supplemented group, 45.0 ± 16.3); however, these values indicate vitamin D deficiency (<50 nmol/L or <20 ng/mL) in both pregnant groups [94,95,96,97]. At 34 gestational weeks, maternal 25(OH)-vitamin D levels were significantly higher in the supplemented group compared to controls (in nmol/L: placebo group, 42.5 ± 20.6; vitamin-D-supplemented group: 66.0 ± 20.4). However, according to the numbers, 1000 IU/day vitamin D supplementation of the pregnant women resulted in only suboptimal (50–75 mmol/L or 20–30 ng/mL) and not optimal (75–125 nmol/L or 30–50 ng/mL) [94,95,96,97] vitamin D status. They observed that CTX showed a two-fold increase from 14 to 34 gestational weeks showing bone resorption in both groups, although this increase was lower in the vitamin-D-supplemented group compared to the placebo group [93]. These results may indicate that vitamin D supplementation may ameliorate and may prevent pregnancy-related reversible bone loss in mothers. In the current literature, there are virtually no human clinical studies that investigate the possible associations between vitamin D deficiency, BMD changes and female fertility and pregnancy. Therefore, further basic and clinical research are needed to explore and map possible connections.

Pregnancy-related transient osteoporosis of the hip (PR-TOH) [98,99] is a rare, benign orthopedic-obstetrical illness; it usually occurs during the third trimester of pregnancy. Although the exact pathophysiology of PR-TOH is still unknown, it is characterized by the appearance of sudden pain in the groin region, the anterior thigh and gluteal area. It may be uni- or bilateral, it may complicate natural vaginal birth, and it may be a non-obstetric indication to perform a cesarean section [98]. It may also be assumed that vitamin D may play a potential role in the pathomechanism of this disease, as the classic skeletal effects of vitamin D on calcium and phosphate homeostasis and bone turnover-metabolism are well known and documented [100]. In addition, the prevalence of vitamin D deficiency in pregnancy is markedly high and is observed to occur on a wide spectrum (8–70%) [101]. Moreover, the content of dietary vitamin–mineral preparations/supplements for pregnant women often vary and do not follow the latest expert recommendations [102]; thus, the dosage may often be inadequate. Therefore, adequate vitamin D supplementation from conception to birth and in postpartum is increasingly important both for the mother and newborn, although currently there are no uniform professional recommendations regarding the exact amount of vitamin D supplementation during pregnancy [103].

**Table 4:** Vitamin D deficiency and bone density in fertile women and pregnancy-related transient osteoporosis of the hip.

Study	Article Type	Study Group	Summary
Ó Breasail et al. [92]	Case control study	Healthy pregnant women (*n* = 53), non-pregnant and non-lactating (*n* = 37)	Significant decrease in volumetric bone mineral density and a different pattern of microarchitecture were seen mainly in the tibia (not in radius) in the pregnant group, suggesting compartment-specific maternal bone mineral density and microarchitecture changes during pregnancy.
Moller et al. [91]	Controlled cohort study	Women planning pregnancy (in total 153 women, conceived *n* = 92), women in postpartum (19 month, *n* = 31) and age-matched controls (*n* = 75)	Pregnancy and breastfeeding also lead to reversible bone loss, which returns to pre-pregnancy levels 19 months after delivery, independent of the length of breastfeeding.
Steib-Furno et al. [99]	Prospective survey and retrospective study	Pregnant women (*n* = 4900)	Pregnancy-related transient osteoporosis of the hip (PR-TOH) is a rare, benign orthopedic–obstetrical illness, it usually occurs during the third trimester of pregnancy.
Curtis et al. [93]	Randomized controlled trial	Pregnant women (Placebo group (*n* = 188), cholecalciferol-supplemented (*n* = 184))	The bone resorption marker urinary C-terminal telopeptide of type I collagen (CTX) increased during pregnancy. This occurred to a lesser extent during vitamin D supplementation during pregnancy and this was inversely associated with maternal bone mass following delivery. These results may indicate that vitamin D supplementation may ameliorate and prevent pregnancy-related reversible bone loss in mothers.

## 7. Comparability of Vitamin D Studies

Publications on vitamin D are increasing exponentially; however, they often present contradictory results. Evaluation of these studies is limited due to small sample sizes, non-randomized patient populations, and on occasion, 25(OH)-vitamin D levels were not measured both at initiation and completion of the trials—in these cases vitamin D levels may not differ in different treatment groups. In other publications, vitamin D supplementation was performed using varying doses for different periods (in many cases, 400 IU, which is the equivalent of a newborn dose) [3]. Ethnicity, latitude and vitamin D ingested via diet and exposure to the sun were not considered. The efficacy of vitamin D supplementation should be studied under well-structured and more detailed circumstances to avoid the abovementioned limitations. These factors may account for the non-significant results found in some of the previous studies.

Currently, vitamin D supplementation is usually determined based on by serum 25(OH)-vitamin D levels. This may be influenced by seasons, latitude, lifestyle, diet, genetic factors and skin pigmentations [3]. There is no consensus regarding the bottom threshold for normal levels; the Endocrine Society and Hungarian guidelines mark this at 30 ng/mL (75 nmol/L) [3,104].

## 8. Summary and Recommendations

Vitamin D demand increases during pregnancy; a minimum of 30 ng/mL or 75 nmol/L vitamin D levels are recommended. In pregnancy, if vitamin D levels are in the normal range (>75 nmol/L), maintenance doses are recommended, while in cases of suspected or proven deficiency, therapeutic doses of vitamin D should be supplemented. Hungarian and Central European guidelines recommend administering 2000 IU vitamin D daily during pregnancy and breastfeeding. In PCOS or in non-PCOS obese patients, 2000–4000 IU daily doses of vitamin D supplementation are recommended [15,96,105]. Obesity and PCOS, short intervals between deliveries, or breastfeeding for a long period pose increased risk factors for vitamin D deficiency.

According to a latest Central and Eastern European Expert Consensus Statement, which provides clinical guidelines in the prevention, diagnosis and treatment of vitamin D deficiency: for women who are planning pregnancy, vitamin D supplementation should start or be maintained as recommended for healthy adults without other risk factors, and the supplementation should be continued during pregnancy and lactation to achieve and maintain optimal vitamin D levels (30–50 ng/mL or 75–125 nmol/L 25(OH)-vitamin D) [106].

At the recommended dose of 2000–4000 IU during pregnancy, vitamin D overdose should not occur. In PCOS (outside the United States, where vitamin D deficiency is rare due to vitamin D supplementation in dairy products), vitamin D deficiency is usually significant enough (usually hundreds of thousands of units of deficiency) that the risk of overdose (with the usual signs of hypercalcemic seizures and central nervous system symptoms, etc.) is limited to extreme doses; however, this has not been encountered in the literature. Using the Coimbra protocol (1000 IU of vitamin D per kg of body weight) may safely be given if accompanied by a strict calcium-poor diet to prevent complications of vitamin D supplementation [107].

## Figures and Tables

**Table 1:** Influence of Vitamin D on fertility and in vitro fertilization.

Study	Article Type	Study Group	Summary
Xu et al. [25]	Cell line (animal)	Secondary preantral follicles were isolated from ovaries of rhesus monkeys	Vitamin D increases survival of the preantral follicle, sustains AMH production and enhances the growth of the antral follicle.
Kinute et al. [21]	Animal Study	VDR null mutant mice (*n* = 3–10 group)	In females: uterine hypoplasia, decreased aromatase activity and aromatase gene expression were observed; in males: decreased sperm count and decreased motility with histological abnormality of the testis were observed.
Akhavizadegan et al. [22]	Case control study	Fertile men (*n* = 116) and infertile men (*n* = 114)	Men with vitamin D levels below 20 ng/mL have significantly lower sperm counts. The mean vitamin D level in the fertile group was significantly higher than in the infertile group.
Dennis et al. [19]	Correlative and intervention study	Mature men (*n* = 113), premenopausal women (*n* = 35), 5- to 6-yr-old boys (*n* = 74)	Serum AMH correlates positively with vitamin D levels in men (but not in boys). Vitamin D levels and AMH levels show a seasonal difference in women, with AMH levels falling by 18% in the winter compared to summer. The change in AMH levels correlates with initial AMH levels and the magnitude of the change in vitamin D levels. Vitamin D supplementation prevents seasonal AMH change.
Ozkan et al. [24]	Prospective Cohort Study	Infertile women undergoing IVF (*n* = 84)	Vitamin D level influences the outcomes of IVF independently of age, BMI, ethnicity and the number of embryo transfers. Vitamin D levels correlate strongly (r = 0.94) with follicular fluid.

**Table 2:** Polycystic ovary syndrome and vitamin D deficiency.

Study	Article Type	Study Group	Summary
Tavakoli et al. [48]	Cell line (human)	Endometrial samples (from women with recurrent spontaneous abortion and healthy controls)	Vitamin D supplementation may have a beneficial effect in case of recurrent miscarriages. As a result of vitamin D supplementation, Th2 cytokine dominance was observed with decreased proliferation of inflammatory cytokines.
Diaz et al. [49]	Cell line (human)	Term placentae samples (37–41 weeks of gestation, from uncomplicated pregnancies)	Calcitriol supplementation prevents the production of TNF alpha, IL-6 and IFN gamma—this is likely mediated by VDR.
Liu et al. [50]	Cell line (human)	Human trophoblastic cell lines from American Type Tissue Culture Collection	In human trophoblast cells, Vitamin D metabolites significantly enhance antibacterial responses.
Kuyucu et al. [34]	Animal study	Prepubertal female rats, control group (*n* = 8), PCOS group (*n* = 8) and PCOS + D3 group (*n* = 8)	Vitamin D treatment significantly reduced endometrial, epithelial and stromal thickness in PCOS patients, as well as pathological proliferation and apoptosis. AMH was also decreased as a result of vitamin D supplementation (this however did not reach the level of significance).
Guo et al. [36]	Case control study	Endometrial samples from women who underwent standardized IVF treatment (*n* = 16)	VDR plays role in the development of endometrial susceptibility: increased VDR expression in the endometrium (especially in the implantation window of the menstrual cycle) is significantly more likely to lead to pregnancy.
Zadeh-Vakili et al. [32]	Case control study	PCOS women (*n* = 260) and women with physiological cycles (*n* = 221)	The genetic variant of VDR is associated with the severity of the clinical features of PCOS, but not with the risk of the disease itself.
Aghadavod et al. [38]	Case control study	Control group (*n* = 20 normal weights and *n* = 20 over-weights); PCOS group (*n* = 20 normal weight and *n* = 20 over-weight)	Vitamin D levels in follicular fluid are significantly lower in PCOS and overweight patients. Vitamin D levels in follicular fluid strongly correlate with BMI. VDR expression in granulosa cells is significantly lower in PCOS/overweight patients than in non PCOS or normal weight individuals.
Zhao et al. [39]	Case control study	In total, 305 women were divided into 4 groups based on serum vitamin D levels.	Optimal vitamin D levels improve embryo quality and lead to significantly higher clinical pregnancy rates.
March et al. [27]	Retrospective birth cohort study	In total, 728 women born between 1973–1975 in a single maternity hospital were traced and interviewed in adulthood (age = 27–34 year; *n* = 728).	Prevalence estimates for Rotterdam and AES may be up two twice of that for NIH criteria A significant proportion of women with PCOS are not diagnosed or are diagnosed late.
Pal et al. [28]	Retrospective cohort study (Secondary analysis of randomized controlled trial data)	Participants in the Pregnancy in PCOS I randomized controlled trial (*n* = 540) who met the National Institutes of Health diagnostic criteria for PCOS.	In women with PCOS, serum vitamin D level is an independent predictor of reproductive success rates following induction of ovulation. Probability of ovulation correlates with vitamin D levels in PCOS. The reproductive threshold for serum vitamin D is higher in than that recommended for the non-pregnant population.
Hahn et al. [45]	Prospective cohort study	PCOS women (*n* = 120)	In PCOS, insulin resistance correlates negatively with vitamin D levels.
Pittas et al. [46]	Randomized controlled trial	Caucasian adults (*n* = 314)	In older healthy adults with impaired fasting glucose (IFG), calcium and vitamin D supplementation may reduce the further development of insulin resistance.

**Table 3:** Vitamin D deficiency and pregnancy.

Study	Article Type	Study Group	Summary
Wilson et al. [74]	Animal study	VDR+/− virgin female mice (*n* = 12) + VDR+/− male mice	In VDR−/− placenta, the expression of Deptor and Prr5 involved in mTOR signaling was decreased significantly.
Cleal et al. [75]	Cell line (human)	Placental samples collected in the Southampton Women’s Survey (*n* = 102)	Maternal vitamin D and vitamin-D-binding protein levels correlate positively with the expression of amino-acid transporter genes of the placenta.
Brodowski et al. [77]	Cell line (human)	Cord blood samples from uncomplicated pregnancies and uncomplicated pregnancy villous placentae	Vitamin D promotes the formation of capillary-like tubules and the migration of endothelial colony-forming cells, minimizing the negative effects of preeclampsia.
Wang et al. [79]	Cell line (human)	Placenta samples from healthy women (38–40 weeks of gestations)	Vitamin D downregulates pro-labor genes such as corticotropin-releasing hormone. This reduces the risk of preterm birth.
Javorski et al. [78]	Cell line (human)	Pregnant women (*n* = 189)	Polymorphisms in the VDR gene (Fok-l and Cdx-2) increase the risk of spontaneous preterm birth.
Wang et al. [80]	Case-control study	GDM women (*n* = 41) and healthy pregnant women (*n* = 40)	Placental CYP24A1 protein and mRNA levels responsible for vitamin D catabolism, VDR protein and mRNA levels are increased in GDM.
Knabl et al. [81]	Case-control study	Placental tissue samples from GDM and control patients (*n* = 40 GDM and *n* = 40 controls)	VDR expression is regulated in a bimodal fashion by calcitriol: high doses (0.1 and 1 nmol/mL) lead to downregulation, while low doses (0.01 nmol/mL) lead to VDR upregulation.
Lacroix et al. [82]	Case-control study	Placental tissue samples from normoglycemic and GDM women.	VDR protein expression was increased in GDM patient placenta samples; Vitamin D decreases IL-6 secretion.
Hou et al. [61]	Case-control study	Placenta and decidua samples were collected following termination of pregnancy (controls *n* = 20 and spontaneous miscarriage *n* = 20)	In cases of spontaneous miscarriage, placental and decidual expression of both the vitamin D receptor and the vitamin-D-binding protein was increased. Women who undergo assisted reproductive technologies should ensure optimal vitamin D levels prior to pregnancy.
Nguyen et al. [72]	Case-control study	Placentae from pregnancies complicated by idiopathic fetal growth restriction (*n* = 25) and gestational-matched controls (*n* = 25)	In cases of idiopathic fetal growth restriction, VDR mRNA and protein levels were all significantly decreased.
Stougaard et al. [58]	Retrospective cohort study	Woman who gave birth between 1983–1988 (*n* = 73,237)	There is no association between increased dietary intake of vitamin D and the incidence of preeclampsia.
Merewood et al. [85]	Retrospective cohort study	Women (*n* = 253) who had undergone a primary cesarean section (*n* = 43)	There is a negative association between maternal serum vitamin D levels and cesarean sections. Women with vitamin D levels below 37.5 nmol/L were almost four times more likely to have a cesarean section.
Lee et al. [51]	Prospective observational study	Women who completed 37 weeks of pregnancy (*n* = 680)	In total, 71.7% of pregnant women were vitamin D deficient, 21.0% of pregnant women were vitamin D insufficient and 7.3% of pregnant women had adequate vitamin D levels.
Xu et al. [53]	Prospective cohort study	Pregnant women (*n* = 827, 101 developed GDM)	Median plasma vitamin D concentrations at the first prenatal visit were significantly lower in women who later developed GMD.
Andersen et al. [64]	Prospective cohort study	Pregnant women (*n* = 1683)	The adjusted hazard of first trimester miscarriage is lower at higher vitamin D levels: vitamin D levels below 50 ng/mL double the risk of a miscarriage. No such relationship was found in the second trimester.
McDonnell et al. [67]	Prospective cohort study	Pregnant women (*n* = 1064)	Maternal vitamin D levels above 40 ng/mL reduce the risk of preterm birth.
Raia-Barjat et al. [76]	Prospective multicenter cohort study	Pregnant women (*n* = 200)	At 32 weeks of gestation, placenta-mediated complications (PMC) were 5-fold higher in vitamin D deficiency patients compared to those with normal vitamin D levels. There is a strong inverse relationship between serum vitamin D levels and the risk of late PMC. Vitamin D plays a role in maintaining placental performance, thus preventing the development of late PMC.
Wang et al. [84]	Prospective case-control study	Mother-infant dyads (36.8 ± 2.7 weeks of gestational, *n* = 125)	In total, 56% of newborns were vitamin D deficient. Neonatal vitamin D deficiency may be associated with winter birth, insufficient sunbathing time, high maternal BMI, insufficient egg consumption, insufficient vitamin D supplementation and adverse health insurance status.
Xiaomang et al. [56]	Randomized controlled trial	Women who had undergone maternity treatment and delivery (*n* = 450)	Vitamin D supplementation may reduce the incidence of preeclampsia while also lowering the IUGR index.
Schulz et al. [59]	Randomized controlled trial	Pregnant women (*n* = 43)	Serum vitamin D levels above 100 ng/mL reduce the expression of soluble Fms-like tyrosine kinase-1 (sFlt-1) and VEGF. Maternal vitamin D supplementation may reduce the transcription of genes that contribute to preeclampsia.
Jefferson et al. [65]	Randomized controlled trial	Pregnant women (*n* = 316)	Vitamin D may have a positive impact on the vaginal microbiome: megasphere correlated negatively and L. crispatus correlated positively with plasma vitamin D levels.
Brooke et al. [87]	Randomized controlled trial	Pregnant women (D-vitamin treated *n* = 59 and control *n* = 67) during the last trimester	The risk of SGA in the control group was almost twice that found in the vitamin-D-supplemented group.
